# Supplementary material for: Mapping the evolution of fertility support policies in China: A content and instrumental analysis
Source: PLoS One. 2025 Oct 9;20(10):e0332137. doi: 10.1371/journal.pone.0332137 (PMC12510515; doi:10.1371/journal.pone.0332137)
Supplement: S1 Appendix — (ZIP) [file pone.0332137.s001.zip › S1 Appendix. 226 original policy documents/30-国家卫生健康委、农业农村部、中国计划生育协会关于服务乡村振兴促进家庭健康行动的实施意见(FBM-CLI-4-336164).doc]

国家卫生健康委、农业农村部、中国计划生育协会关于服务乡村振兴促进家庭健康行动的实施意见

**发布部门：** [国家卫生健康委员会](https://www.pkulaw.com/law/chl?Aggs.IssueDepartment=60321) [农业农村部](https://www.pkulaw.com/law/chl?Aggs.IssueDepartment=60240) [中国计划生育协会](https://www.pkulaw.com/law/chl?Aggs.IssueDepartment=70572) [机构沿革](javascript:void(0))

**发文字号：**国卫人口发〔2019〕53号

**发布日期：**2019.09.04

**实施日期：**2019.09.04

**时效性：** [现行有效](https://www.pkulaw.com/law/chl?Aggs.TimelinessDic=01)

**效力级别：** [部门规范性文件](https://www.pkulaw.com/law/chl?Aggs.EffectivenessDic=XE0302)

**法规类别：** [人口计划管理](https://www.pkulaw.com/law/chl?Aggs.Category=09701)

国家卫生健康委、农业农村部、中国计划生育协会关于服务乡村振兴促进家庭健康行动的实施意见
（国卫人口发〔2019〕53号）

各省、自治区、直辖市及新疆生产建设兵团卫生健康委、农业农村厅（局）、计生协：
　　根据《中国共产党农村工作条例》《中共中央 国务院[关于实施乡村振兴战略的意见](https://www.pkulaw.com/chl/a6b7e29eb561b45dbdfb.html)》《“健康中国2030”规划纲要》和《中共中央办公厅 国务院办公厅关于加强和改进乡村治理的指导意见》的有关要求，为树立大卫生大健康理念，进一步发挥计生协作用，深入开展家庭健康促进行动，全面服务乡村振兴，特制定本实施意见。
　　**一、**指导思想和基本原则
　　（一）指导思想。以习近平新时代中国特色社会主义思想为指导，深入贯彻党的十九大和十九届二中、三中全会精神，落实习近平总书记对计生协工作的重要指示精神，按照实施乡村振兴战略、健康中国战略的总体要求，以人民为中心，坚持问题导向，突出体制机制创新，引导广大农村群众重视家庭健康，鼓励人人参与、人人尽力、人人享有，全面提升群众健康素养，着力建设新时代乡村家庭婚育文明，营造良好健康氛围，为建设充满活力、和谐有序的乡村社会奠定坚实基础。
　　（二）基本原则。
　　坚持正确方向。加强政治引领和思想引导，把坚持党的领导和团结服务广大群众有机统一起来，自觉融入乡村振兴战略，主动对接乡村振兴重大工程、重大计划，引导广大农村群众听党话、感党恩、跟党走，为全面实现农业强、农村美、农民富贡献力量。
　　坚持服务群众。顺应亿万农民对美好生活的向往，以真诚倾听群众呼声，真实反映群众愿望，真情关心群众疾苦为出发点和落脚点，拓宽服务领域，丰富服务内容，创新服务载体，充分调动农村群众的积极性、主动性和创造性，汇聚起投身乡村振兴、促进家庭健康的强大力量。
　　坚持多元共治。深刻把握现代化建设规律和城乡关系变化特征，聚焦乡村治理中的突出矛盾和主要问题，配合党委政府协调激活多元主体，协同整合各类资源，既尽力而为又量力而行，推动形成丰富多彩、适合本地特点的具体实践，促进共建共治共享乡村治理的新局面。
　　坚持基层基础。坚持眼睛向下、重心下移，把更多服务资源向基层倾斜，创新基层组织设置、会员发展、活动开展的方式方法，把组织覆盖与工作覆盖有机结合起来，夯实工作基础；鼓励先行先试、大胆探索，做到顶层设计源自基层、创新成果惠及基层。

　　**二、**主要任务
　　（一）加强农村家庭健康教育服务，普及群众健康生活。适应新时代卫生健康工作由“以治病为中心”向“以人民健康为中心”转变的要求，发挥计生协的组织网络优势、群众工作优势，参与提供全方位全周期的群众性健康服务，助推健康治理的重心落到基层。鼓励各地加强对计生协骨干的卫生健康知识和技能培训，培训合格后择优纳入家庭医生签约服务团队，从事基本公共卫生服务等工作。通过开播家庭健康大讲堂、设立健康宣传栏、评选健康家庭等，引导群众把预防放在优先位置，树立“群众是健康第一责任人”的理念，倡导健康生活方式，普及合理膳食、心理健康、意外伤害预防、“三减三健”等知识。实施农村妇女生殖健康项目，宣传普及育龄群众生殖健康知识，提供生殖健康咨询和生育调节指导、随访和转介等服务，提高群众生殖健康水平。开展青少年性与生殖健康教育，帮助农村地区青少年树立阳光健康的生活态度，降低青少年怀孕率和人工流产率，减少意外妊娠和性病艾滋病传播。
　　（二）深入开展幸福家庭创建活动，塑造社会主义家庭文明新风尚。加强家庭、家教、家风建设，巩固家庭抚幼养老功能，弘扬尊老爱幼、男女平等、夫妻和睦、勤俭持家、邻里团结等传统美德，引领良好家风家规。依托人口学校、人口文化大院、农家书屋和文艺宣传队等，推动形成爱国爱家、相亲相爱、向上向善、共建共享的社会主义家庭文明新风尚。完善村规民约，将“婚育文明”融入文明公约、家规家训，自觉抵制封建迷信，破解农村大操大办、高额彩礼、铺张浪费等陈规陋习，推动移风易俗。开展“幸福家庭”“好婆婆好媳妇”“好邻居”等评选活动，突出典型示范引领作用，引导群众积极创建幸福家庭，使千千万万个家庭共同为促进家庭和睦、亲人相爱、下一代健康成长、老年人老有所养而努力。
　　（三）拓展深化生育关怀，真情服务“一老一小”。针对计划生育特殊家庭面临的困难和问题，完善精神慰藉、走访慰问、志愿服务、保险保障四项制度，规范提供心理疏导、精神抚慰、生活帮扶、保险保障等多元化服务，实现联系人制度、就医“绿色通道”和家庭医生签约服务“三个全覆盖”，让计划生育特殊家庭感到在社会上有地位、精神上受抚慰、经济上得实惠。农村低保、脱贫攻坚及其他优惠政策向计划生育特殊家庭倾斜，体现优先优待。开展“优生优育进万家”主题宣传活动，普及孕产期和育儿期健康等知识，提高家庭科学育儿能力。积极参与疾病预防和出生缺陷干预活动，开展“幸福微笑--救助唇腭裂儿童”等公益项目。巩固和优化“会员之家”功能，有条件的建立优生优育指导中心等活动阵地，帮助解决农村婴幼儿照护、儿童早期发展难题，为群众按政策生育创造更好条件。积极参与农村留守儿童、留守妇女、留守老年人关爱服务体系，加强疾病防治、医疗救助、健康教育和咨询、生产生活帮扶、信息采集和强制报告等工作。
　　（四）搭建帮扶关爱平台，切实改善困难家庭生活。深入开展计生“三结合”、小额贴息贷款、幸福工程、金秋助学等特色项目，帮助计划生育困难家庭脱贫，改善困难家庭福祉。深入实施计划生育保险等商业保险项目，探索开发符合群众需求的保险产品，增强计划生育家庭防范和抵御风险的能力。落实计生协骨干会员联系户制度，构建专业化、网格化、个性化的志愿者服务体系，推动骨干会员和志愿者与困难家庭结对帮扶，帮助他们早日过上幸福生活。
　　（五）推动计划生育基层群众自治转型升级，促进农村社会和谐。完善计划生育自治章程的内容、重点和工作方式，推动计生基层群众自治“源于计生又超越计生”，维护农村社会和谐稳定。跟踪了解计划生育等相关法律政策的落实情况和群众的迫切需求，及时反映群众诉求，推动完善相关法律政策。组织群众依法修订村民自治章程或村规民约，引导群众自我管理、自我教育、自我服务，健全矛盾纠纷多元化解机制，切实维护群众合法权益。支持和鼓励计生协组织融入自治、法治、德治相结合的乡村治理体系，积极参与美丽乡村建设、农村信用体系建设、扫黑除恶专项斗争等工作，汇聚乡村善治的合力。

　　**三、**保障措施
　　（一）加强组织领导。各级卫生健康行政部门、农业农村行政部门和计生协组织要高度重视，积极争取各级党委政府的支持，在实施本级乡村振兴规划中统筹推进家庭健康促进行动。各级卫生健康行政部门要加大政策支持，提供有力保障，爱国卫生、健康扶贫、基层卫生、妇幼健康、疾病预防控制、宣传等机构要积极履职尽责，推进健康中国建设，服务乡村振兴战略。各级计生协要围绕地方党委政府的工作部署，因地制宜制订实施方案及路线图、任务书，抓好工作落实。
　　（二）完善工作机制。各级卫生健康行政部门、农业农村行政部门、计生协组织要统筹协调本部门的相关资源，强化政策、力量配备，建立定期会商工作机制，加强工作指导。切实履行好投身乡村振兴、促进家庭健康行动的牵头部门责任，密切配合，形成合力，将各项工作任务落在实处。统筹使用好相关经费，加大对贫困地区和边境地区的倾斜力度，加强经费使用情况的绩效评估，确保专款专用和使用效果。鼓励企事业单位、社会组织等社会资源支持，吸引社会资本投入。
　　（三）做好总结宣传。总结推广可复制的经验做法，确保各项任务落到实处、取得实效。加强工作宣传，注重线上线下结合，运用好全媒体手段，宣传并激励表扬有突出贡献的组织和个人，营造良好工作氛围。

卫生健康委
农业农村部
中国计生协会
2019年9月4日

© 北大法宝：（[www.pkulaw.com](https://www.pkulaw.com/)) 专业提供法律信息、法学知识和法律软件领域各类解决方案。北大法宝为您提供丰富的参考资料，正式引用法规条文时请与标准文本核对。
欢迎查看所有[产品和服务](https://www.pkulaw.com/helps/2.html)。法宝快讯：[如何快速找到您需要的检索结果？](https://www.pkulaw.com/helps/70.html)[法宝](https://www.pkulaw.com/helps/69.html)V6有何新特色？

扫描二维码阅读原文

**原文链接：**<https://www.pkulaw.com/chl/bb67a84763a51170bdfb.html>
